# Digital-assisted multidisciplinary treatment for complex occlusal rehabilitation: an 18-month follow-up case report

**DOI:** 10.1186/s12903-024-04574-2

**Published:** 2024-07-18

**Authors:** Anna Qiu, Lixia Xu, Yuan Zhang, Kailun Chen, Sheng Fang, Ying Zhang, Liangjun Zhong, Rui He

**Affiliations:** 1 Center of Stomatology, The Affiliated Hospital of Hangzhou Normal University, No. 126 Wenzhou Road, Hangzhou, Zhejiang China; 2 School of Stomatology, Hangzhou Normal University, No. 120, Jinhua Road, Hangzhou, Zhejiang China; 3 The 3rd People’s Hospital of Deqing, Huzhou, Zhejiang China

**Keywords:** Occlusal rehabilitation, Dentition defect, Multidisciplinary treatments, Digital technology, Dental occlusion

## Abstract

**Background:**

This case report highlights the importance of standardized and fully digital sequential treatment in complex occlusal rehabilitation cases. To fully resolve the patient’s dental needs, such cases often require multidisciplinary interventions including periodontal therapy, endodontic treatment, anterior esthetics, implant restoration, and prosthetic rehabilitation. A fully digital workflow (including facial scanners, intraoral scanners, jaw motion tracking systems, virtual articulators, and computer-aided design software) streamlined the complex treatment, enhancing workflow simplicity, efficiency, visibility, and precision.

**Case presentation:**

The patient presented with decreased chewing efficiency of the upper and lower prostheses, along with unsatisfactory esthetic appearance of the anterior teeth. After physical examination and radiological assessment, this complex occlusal rehabilitation case required periodontal therapy, anterior esthetic enhancement, implant restoration, and fixed prosthetic rehabilitation. Therefore, a fully digital workflow was adopted. Full-crown prostheses were placed on teeth 13, 23, and 34; a fixed bridge encompassed positions 32 to 42, and single implant crowns were placed on teeth 35 and 36. Implant-supported fixed bridges were constructed for teeth 12 to 22 and 44 to 46, anchored by implants at teeth 12, 22, 44, and 46. All definitive prostheses were fabricated from zirconia ceramics, chosen for their durability and esthetic characteristics. Finally, restorations with satisfactory esthetic and functional characteristics were seated, preserving the tooth and its supporting structures. During treatment and follow-up, the T-scan occlusal analysis system was utilized to continuously monitor and guide the adjustment of occlusal distribution across the patient’s dental arches. After 18 months, the patient remains satisfied with the definitive restorations.

**Conclusions:**

This report is intended to help dentists understand and implement standardized and fully digital workflows during the management of complex occlusal rehabilitation cases; it may also facilitate harmonious integration of esthetic and functional characteristics.

## Background

Prosthodontics is not practiced in isolation, particularly in cases of complex occlusal rehabilitation, because many patients have various dental needs or medical health issues for which multidisciplinary treatment is warranted [1]. Complex occlusal rehabilitation poses a substantial challenge, requiring a meticulous and integrated workflow [2, 3]; multidisciplinary treatment is typically necessary to achieve the best outcome. This process demands a comprehensive approach that involves periodontal therapy, endodontic treatment, anterior esthetics, implant restoration, and prosthetic rehabilitation [4–6].

In the conventional workflow, each step—from precise impression-taking and accurate model pouring to the meticulous transfer of occlusal relationships onto a conventional articulator—has an important role [7]. Deviations within this workflow can compromise the precision and accuracy of the overall rehabilitative procedure [8]. Conventional articulators, which rely on data derived from population averages, may have limitations when applied to individual cases [9, 10]. Furthermore, a lack of communication among dentists, technicians, and patients can introduce prognostic uncertainty and impede the achievement of optimal esthetic outcomes [11].

A digital workflow helps to achieve a predictable and generally favorable prognosis. The integration of digital data from facial scanners, intraoral scanners, jaw motion tracking systems, virtual articulators, and computer-aided design (CAD) software enables the creation of a virtual dental patient to assist with therapy and design. This approach offers greater visibility and efficiency [12–15]. In complex cases that involve extensive oral esthetic defects and require multidisciplinary treatment, a fully digital workflow is useful for providing comprehensive diagnoses and designs. This workflow achieves optimal relationships between the lips and teeth, as well as between the teeth and gums [6]. It also enables precise design of the virtual dentition, including the occlusal vertical dimension, centric relation (CR), target arch shape, smile appearance, dynamic occlusion, and static occlusion [16, 17].

This report describes the planning and fabrication of restorations for occlusal rehabilitation combined with a fully digital workflow after multidisciplinary therapy.

## Case presentation

**Fig. 1:**
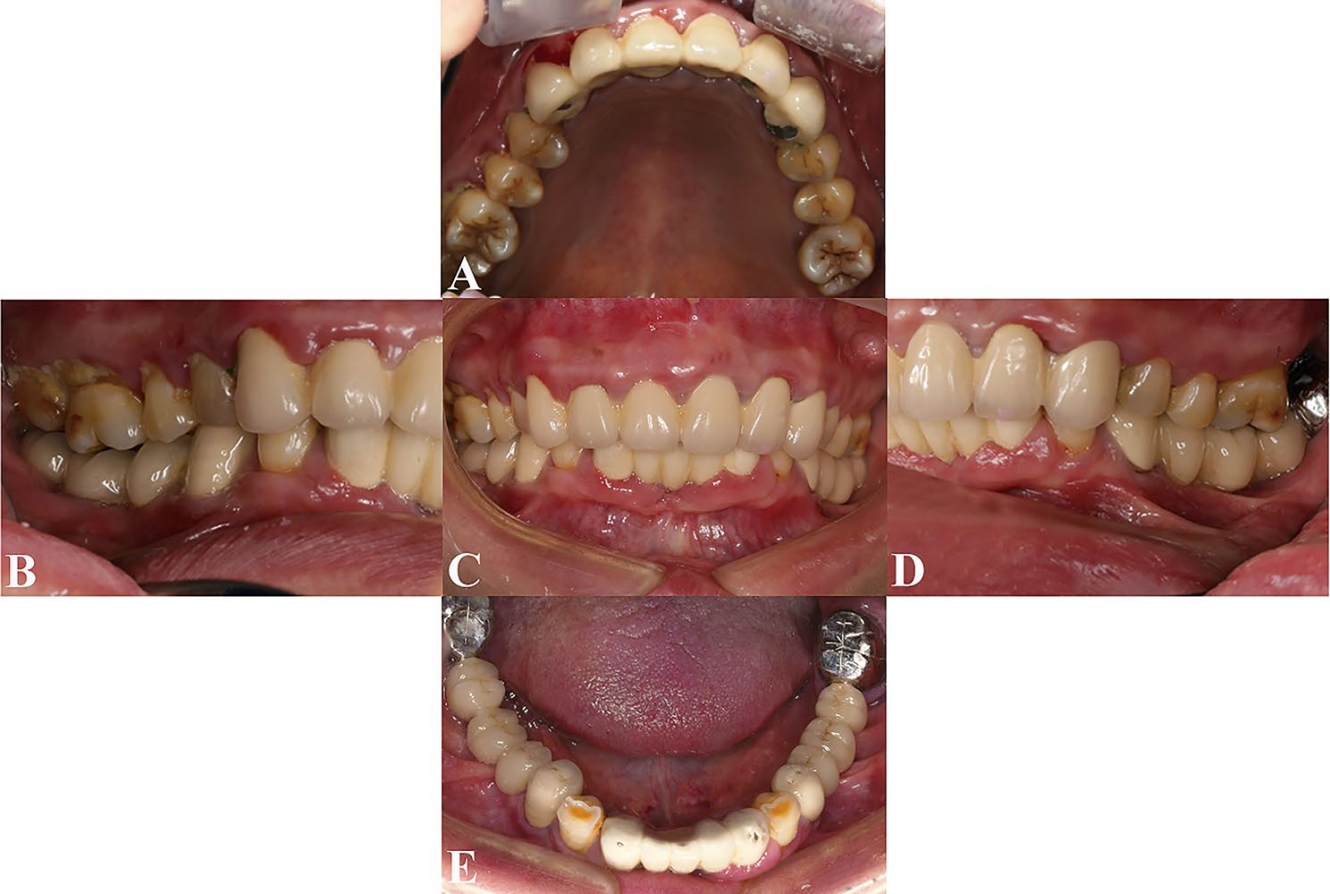
Initial intraoral photographs revealed poor oral hygiene. **A**, Maxillary occlusal view. **B**, Right buccal view. **C**, Frontal view. **D**, Left buccal view. **E**, Mandibular occlusal view

**Fig. 2:**
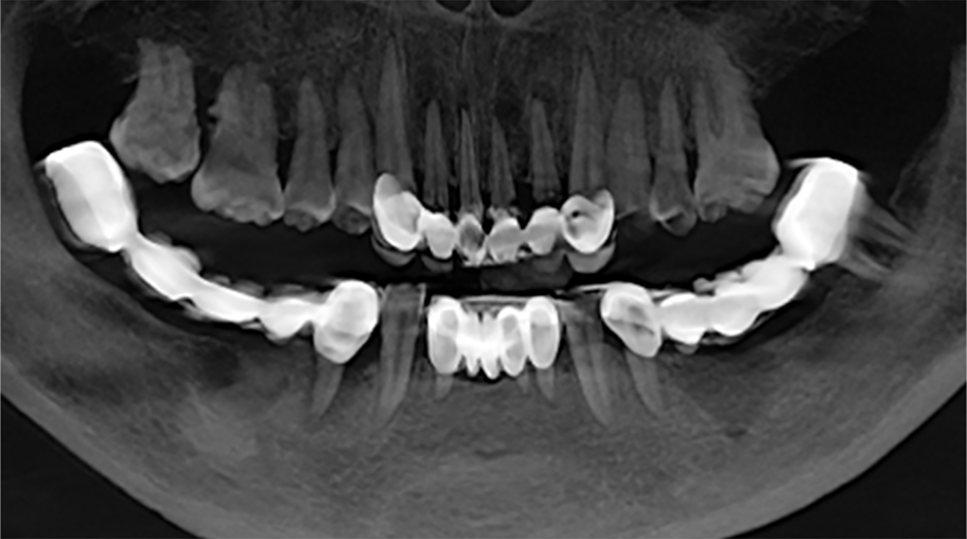
Pre-treatment dental panoramic radiography showed apical cysts in the anterior maxilla and almost complete resorption of the right mandibular last molar

A 55-year-old woman initially presented to the Center of Stomatology at the Affiliated Hospital of Hangzhou Normal University with the chief complaints of decreased chewing efficiency of the upper and lower prostheses, along with unsatisfactory esthetic appearance of the anterior teeth; these problems had developed during the previous year. Other than hypertension, the patient’s medical history was unremarkable; no systemic illnesses or relevant family history were reported. Clinical examination revealed poor oral hygiene, characterized by visible food debris and calculus. The gums were red and swollen, with an obvious lack of gingival attachment and an abnormally high buccal frenum attachment in the posterior mandible. The patient exhibited missing teeth at positions 27, 35 to 37, 41, and 45 to 48. Defective fixed prostheses were present in both the anterior and posterior mandible, as well as the anterior maxilla. The distal-extension fixed bridge encompassing positions 44 to 48 demonstrated moderate mobility. Furthermore, an uneven occlusal curve was observed, contributing to malocclusion and reduced masticatory efficiency (Fig. 1). Panoramic radiography revealed approximately 2–4 mm apical cysts associated with the four maxillary incisors, periapical inflammation around teeth 13 and 23, and clinically significant loss of supporting tissue around tooth 44 (Fig. 2).

The patient provided verbal informed consent prior to treatment initiation. The treatment plan included endodontic, implantology, periodontic, and prosthodontic treatments. The initial mandibular motion trajectory was recorded using a jaw motion tracking system (JMA, Zebris; Isny, Germany). Because the patient’s erratic temporomandibular joint motion severely hindered lateral movement, only the personalized sagittal condyle inclination was used to guide the design of the restorations. After removal of the original prostheses, visible defects were observed in teeth 12, 21, and 22, along with subgingival margins. Thus, two treatment options were presented to the patient. Option A involved extraction of the four maxillary incisors followed by apical surgery, followed by placement of implant-supported bridges for teeth 12 to 22; the bridges would be anchored by implants at teeth 12 and 22. Option B involved endodontic treatment of the four maxillary incisors, followed by apical surgery and crown lengthening; a fixed bridge would be placed encompassing teeth 12 to 22. The patient selected option A due to financial constraints and concerns about the prognosis of the fixed bridge (e.g., apical surgery and crown lengthening lead to shorter roots). Tooth 44 had undergone substantial structural loss and exhibited severe mobility. Ultimately, we extracted teeth 11, 12, 21, 22, 38, and 44; we performed endodontic treatment on teeth 13, 23, and 34. Furthermore, we performed scaling and root planing, then eliminated occlusal trauma factors to preserve the remaining periodontal tissues.

Three months later, implant surgery was conducted in stages. Cone-beam computed tomography scans were used for meticulous evaluation of dental implant positions. Six implants were placed to replace teeth 12, 22, 35, 36, 44 and 46 (ITI Implants, Straumann AG; Waldenburg, Switzerland; 12 and 22: ∅ 3.3 mm NC, SLA 10 mm; 35, 36, and 44: ∅ 4.1 mm RC, SLA 10 mm; 46: ∅ 4.1 mm RC, SLA 8 mm). Due to insufficient bone mass, Bio-Gide was placed for periosteal augmentation and Bio-Oss was placed for bone grafting (Geistlich Pharma; Wolhusen, Switzerland) on the buccal aspect of the maxillary anterior region.

**Fig. 3:**
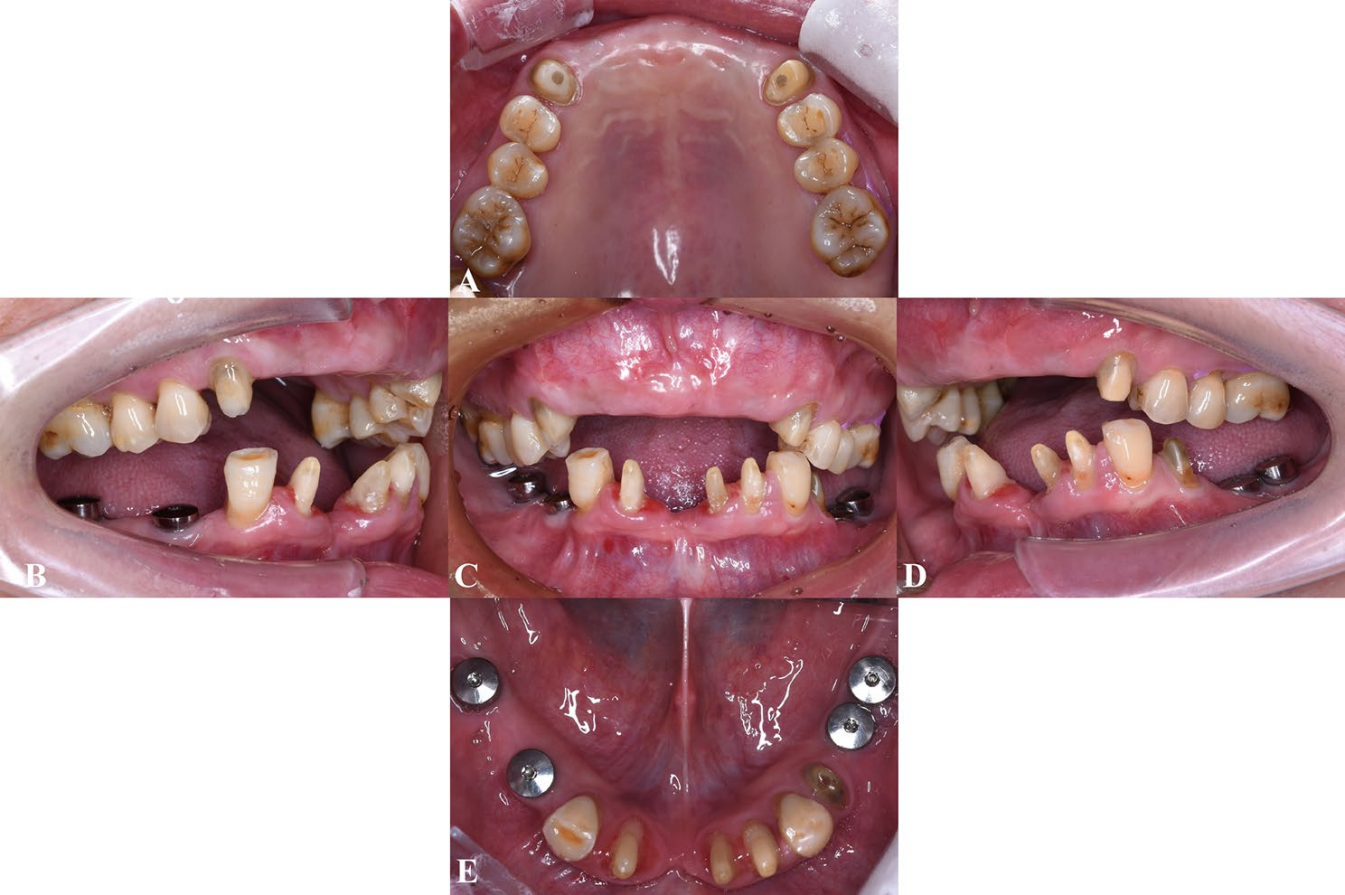
Intraoral views after implant surgery and tooth preparation. **A**, Maxillary occlusal view. **B**, Right buccal view. **C**, Frontal view. **D**, Left buccal view. **E**, Mandibular occlusal view

Prosthetic restorations were scheduled for 6 months later (Fig. 3). Because of financial constraints, the patient chose not to restore tooth 47; she also limited the restoration to the first molars on the upper and lower left sides.

To facilitate treatment planning, a diagnostic scan and interocclusal record were captured using an intraoral scanner (TRIOS 3, 3Shape; Copenhagen, Denmark). A three-dimensional (3D) facial scan was obtained using a transfer fork with a 3D facial scanner (3D face, Jijia; Shanghai, China). A jaw motion tracking system was utilized to track and record mandibular motion, including lateral, protrusive, and opening movements, as well as mastication. These data were merged to create a virtual dental patient.

**Fig. 4:**
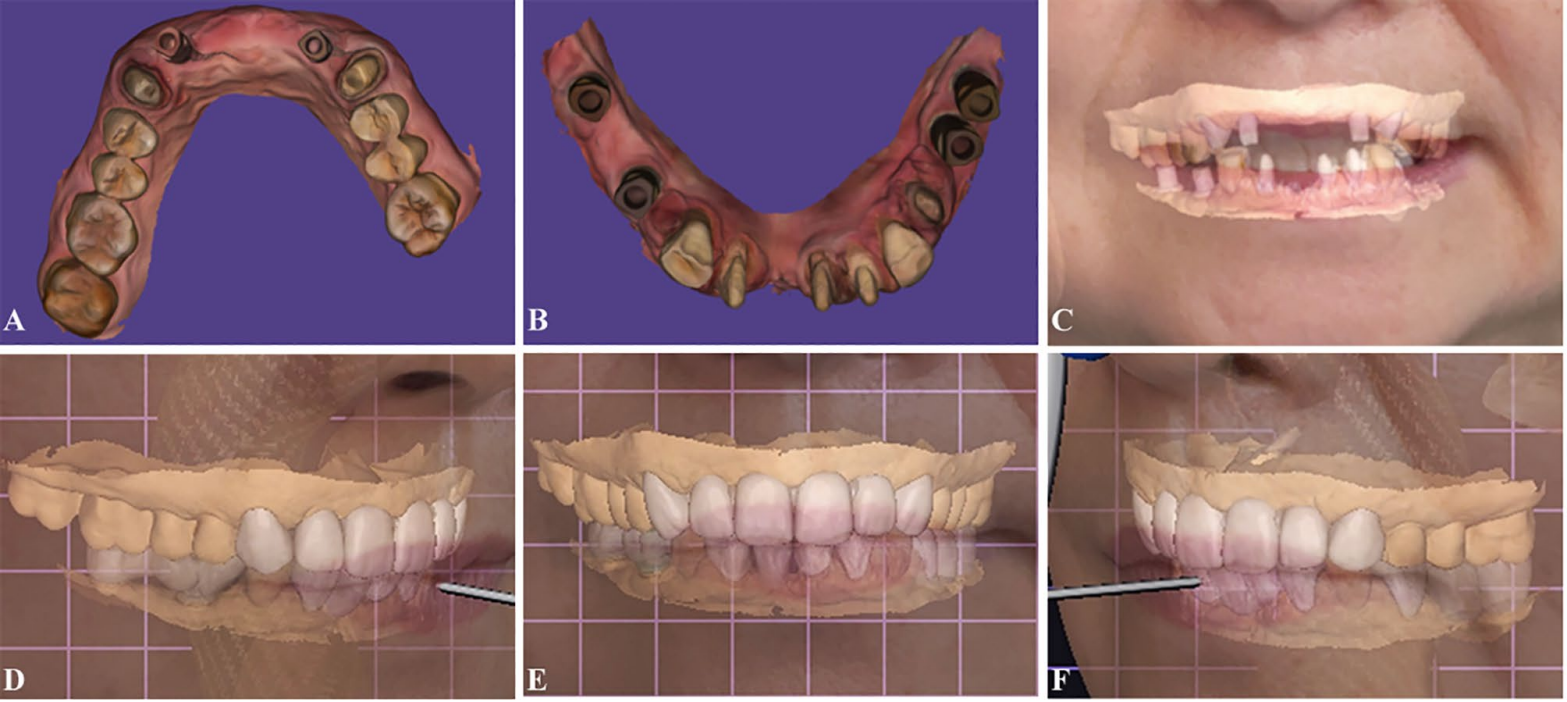
Designs of interim restorations in CAD software (Exocad). **A**–**B**, Intraoral scans of maxillary and mandibular tooth preparations and implants. **C**, 4D virtual patient created by CAD-CAM software combined with intraoral scanning, facial scanning, and JMT systems. **D**–**F**, Virtual patient with interim restorations

The mandibular curve, developed based on natural teeth in the posterior maxilla, aided in the rehabilitation of occlusion. Then, the CR was established based on the recorded mandibular movement trajectory, ensuring that restorations demonstrated appropriate fit. Interim restorations were planned by CAD software (Fig. 4), milled (CEREC MC XL; Dentsply Sirona) from composite resin blocks, and seated.

**Fig. 5:**
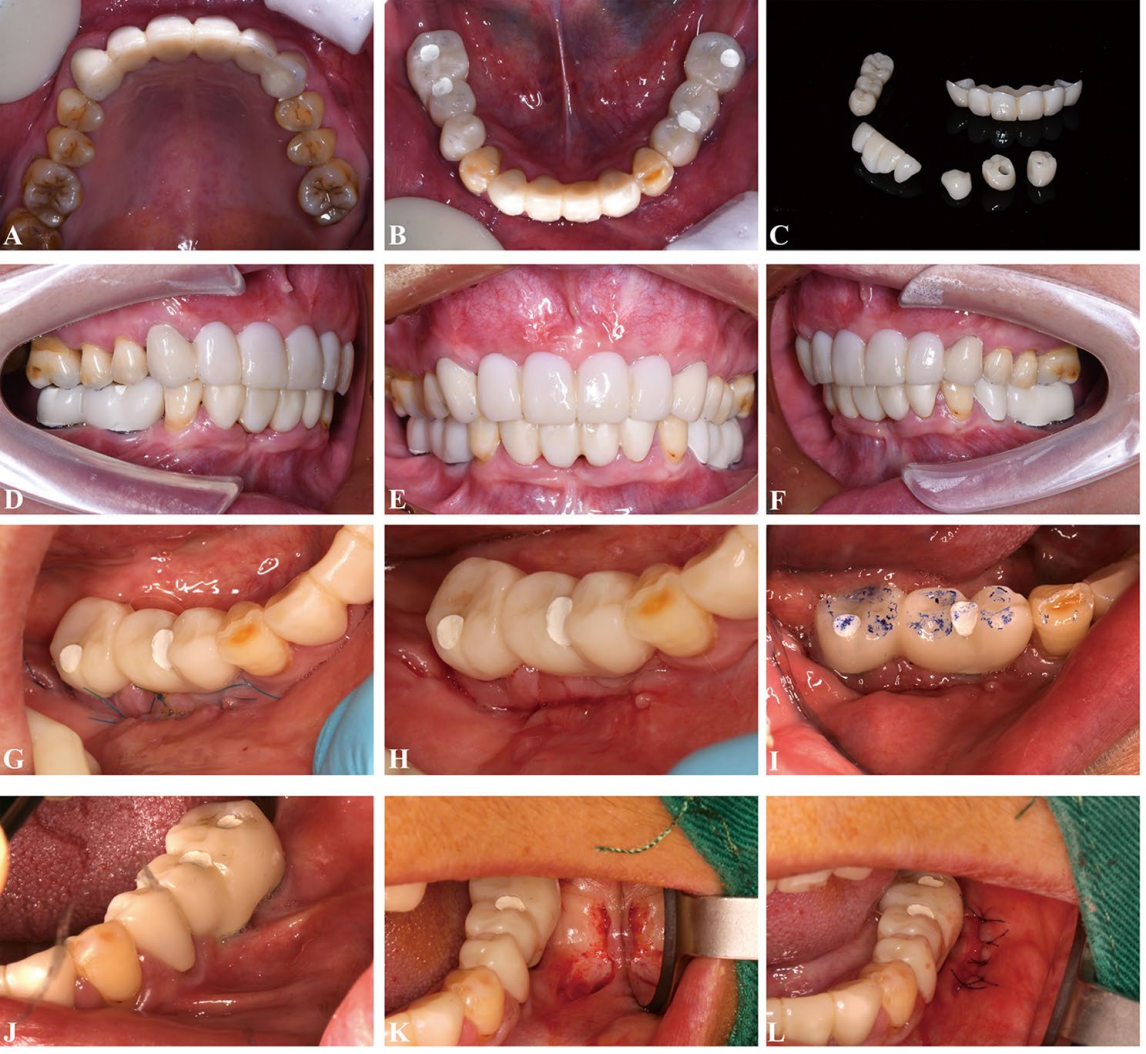
**A**–**F**, Intraoral views of interim restorations. **G**–**I**, Apically repositioned periodontal flap surgery. **J**–**L**, Buccal frenotomy

The interim restorations were worn for 3 months to ensure that a suitable and stable occlusal relationship had been achieved. During this period, apically repositioned periodontal flap surgery was performed on the right posterior mandibular implants to increase the width and thickness of attached gingiva in this region. The buccal aspect was covered with Mucograft^®^ (Geistlich Pharma; Wolhusen, Switzerland). The left buccal mandibular frenum was subjected to frenotomy; this procedure was intended to prevent gingival tension and reduce bone resorption around the implants (Fig. 5).

**Fig. 6:**
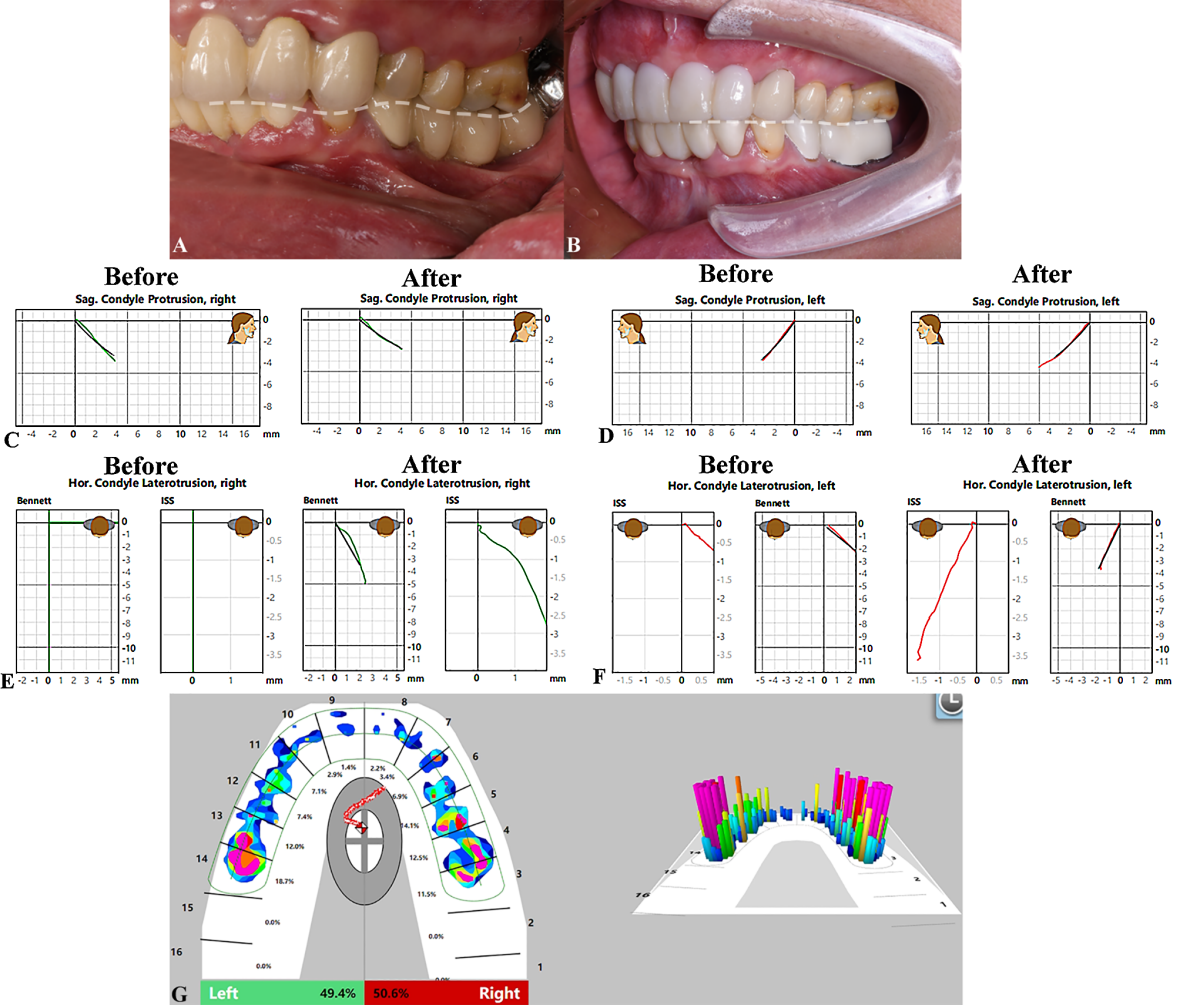
**A** White dashed line represents the occlusal curve. An uneven occlusal curve was evident before treatment. **B**, The reconstructed curve is smooth and continuous. **C**–**F**, Mandibular movement trajectory records. The new trajectory is symmetrical and stable. **G**, T-scan recording. The distribution of biting pressure is symmetrical

Before placement of the definitive restorations, the mandibular movement trajectory was checked and the T-scan tool (Tekscan, Inc.; South Boston, MA, USA) was used to confirm that the stomatognathic system was healthy and functional. The new trajectory differed from the previously recorded trajectory (Fig. 6).

**Fig. 7:**
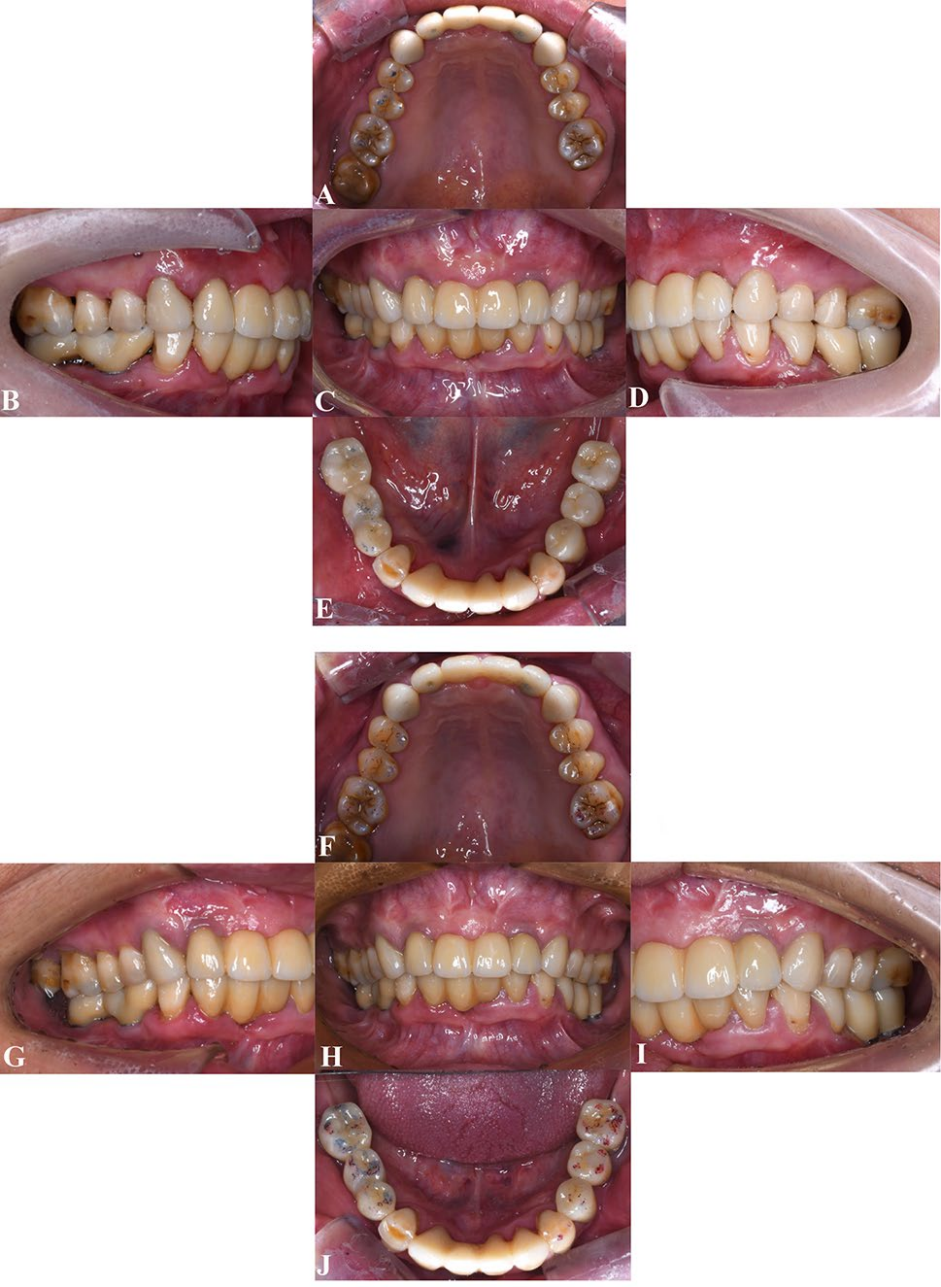
Intraoral views of the definitive restorations showing excellent integration. **A**, Maxillary occlusal view. **B**, Right buccal view. **C**, Frontal view. **D**, Left buccal view. **E**, Mandibular occlusal view. **F**–**J**, Intraoral views at the 18-month follow-up visit. **F**, Maxillary occlusal view. **G**, Right buccal view. **H**, Frontal view. **I**, Left buccal view. **J**, Mandibular occlusal view

On the basis of the interim restorations, the occlusal relationships were rescanned for the definitive restorations, which were then fabricated and seated. Full-crown prostheses were placed on teeth 13, 23, and 34; a fixed bridge encompassed positions 32 to 42, and single implant crowns were placed on teeth 35 and 36. Implant-supported fixed bridges were constructed for teeth 12 to 22 and 44 to 46. All definitive prostheses were fabricated from zirconia ceramics (DD cubeX², Dental Direkt GmbH; Spenge, Germany), chosen for their durability and esthetic characteristics. All implant prostheses were cement-retained with cemented abutments. (Variobase abutments, Straumann AG; Waldenburg, Switzerland; 12 and 22: NC, 5.5 mm; 35, 36, 44, and 46: RC, 5.5 mm). At the 18-month follow-up visit, the patient remained satisfied with the definitive restorations (Fig. 7).

## Discussion and conclusions

The conventional workflow primarily focuses on the patient’s dentition, utilizing static dental information to guide treatment and restoration approaches [8]. However, the stomatognathic system comprises a coordinated set of functional tissues; static dental information alone is insufficient for comprehensive and realistic simulation of a patient’s condition [18–20]. The development of digital technologies in recent years has enabled the acquisition of dynamic 3D data representing multiple tissues and organs. Upon integration of these dynamic data, a four-dimensional (4D) virtual patient can be constructed; this virtual patient seamlessly combines static esthetics with dynamic functionality, offering a more holistic and realistic approach to dental care [21].

Virtual dental patients facilitate the virtual design of interim restorations by incorporating facial features. This ability is particularly beneficial for anterior esthetics because patients can directly visualize their smile appearance after the restoration has been seated, greatly enhancing communication among dentists, technicians, and patients [22].

Building on these advancements, the jaw motion tracking system accurately simulates the patient’s anatomical movement trajectory, thereby assisting with CR identification in a manner that is more precise than the average values used in a conventional tracing articulator [17]. This approach allows the restoration to be integrated with the facial architecture and its major reference points, facilitating accurate establishment of the CR and virtual modification of vertical occlusion. The integration of a virtual articulator with dental CAD systems optimizes the calibration of prosthetic occlusal profiles, ensuring harmonious and functional occlusal design [18]. Furthermore, correct occlusion must be established to facilitate the long-term maintenance of prostheses with and without implant support. This approach can substantially reduce chairside time for occlusal adjustments and enhance patient acceptance.

The T-scan occlusal analysis system is a computerized technology that uses paper-thin, flexible, and pressure-sensitive sensors to record the distribution of occlusal contacts [23]. This system enables dentists to view patients’ occlusal contacts and associate them with specific teeth, providing a detailed analysis of occlusal force distribution within restorations [24]. It records key parameters such as the center of force, demonstrating occlusal force symmetry; first contact, the area of early contact between maxillary and mandibular teeth, etc. By ensuring an even distribution of occlusal forces, the T-scan tool helps to prevent excessive stress on any part of the dental arch, thereby protecting the stomatognathic system [25], enhancing the longevity and stability of restorations, and greatly contributing to the success of rehabilitation efforts [26]. The combination of the T-scan findings and impressions from bite registration paper is important to ensure a strong and stable occlusal relationship [27].

Periodontal surgery and oral hygiene education were conducted to maintain the long-term health of the restoration [28]. Insufficient gingival attachment around the implant can impede plaque control [29]. Gingival attachment plays a key role in maintaining peri-implant health and preventing dental implant infections [30]. When the soft tissue had stabilized, definitive restorations were seated.

This complex occlusal rehabilitation case required a comprehensive and multidisciplinary treatment approach involving periodontal therapy, anterior esthetic enhancement, implant restoration, and fixed prosthetic rehabilitation.

A fully digital workflow allowed comprehensive evaluation of the patient, thereby establishing a holistic perspective for diagnosis and treatment. The application of digital technology greatly enhanced patient comfort and acceptance, avoided distortion of the impression material, allowed pre-visualization of restorative outcomes, and offered potential cost and time savings by minimizing the need for repeat treatments. Finally, restorations with satisfactory esthetic and functional characteristics were seated, preserving the tooth and its supporting structures. After 18 months, the patient remains satisfied with the definitive restorations.

## Data Availability

All data supporting the findings is contained within the manuscript.

## Abbreviations

CAD: Computer-Aided Design
CAD-CAM: Computer Aided Design-Computer Aided Manufacturing
CR: Centric Relation
3D: Three-Dimensional
4D: Four-Dimensional
JMT: Jaw Motion Tracking
